# Comparative one-factor-at-a-time, response surface (statistical) and bench-scale bioreactor level optimization of thermoalkaline protease production from a psychrotrophic *Pseudomonas putida *SKG-1 isolate

**DOI:** 10.1186/1475-2859-10-114

**Published:** 2011-12-28

**Authors:** Santosh K Singh, Sanjay K Singh, Vinayak R Tripathi, Sunil K Khare, Satyendra K Garg

**Affiliations:** 1 Center of Excellence, Department of Microbiology, Dr. Ram Manohar Lohia Avadh University, Faizabad-224001, UP, India; 2 Department of Chemistry, Indian Institute of Technology, Hauz-Khas, New Delhi-110016, India

## Abstract

**Background:**

Production of alkaline protease from various bacterial strains using statistical methods is customary now-a-days. The present work is first attempt for the production optimization of a solvent stable thermoalkaline protease by a psychrotrophic *Pseudomonas putida *isolate using conventional, response surface methods, and fermentor level optimization.

**Results:**

The pre-screening medium amended with optimized (w/v) 1.0% glucose, 2.0% gelatin and 0.5% yeast extract, produced 278 U protease ml^-1 ^at 72 h incubation. Enzyme production increased to 431 Uml^-1 ^when Mg^2+ ^(0.01%, w/v) was supplemented. Optimization of physical factors further enhanced protease to 514 Uml^-1 ^at pH 9.0, 25°C and 200 rpm within 60 h. The combined effect of conventionally optimized variables (glucose, yeast extract, MgSO_4 _and pH), thereafter predicted by response surface methodology yielded 617 U protease ml^-1 ^at glucose 1.25% (w/v), yeast extract 0.5% (w/v), MgSO_4 _0.01% (w/v) and pH 8.8. Bench-scale bioreactor level optimization resulted in enhanced production of 882 U protease ml^-1 ^at 0.8 vvm aeration and 150 rpm agitation during only 48 h incubation.

**Conclusions:**

The optimization of fermentation variables using conventional, statistical approaches and aeration/agitation at fermentor level resulted in ~13.5 folds increase (882 Uml^-1^) in protease production compared to un-optimized conditions (65 Uml^-1^). This is the highest level of thermoalkaline protease reported so far by any psychrotrophic bacterium.

## Background

Proteases are one of the most exploited industrial groups of enzymes accounting for nearly 60% of the total worldwide sale of enzymes [1]. In order to meet the growing demand of proteases with cost effectivity, scaling-up of the industrial process is imperative. Joo et al. [2] opined that 30-40% cost of industrial enzymes depends on the growth medium. Major nutritional factors affecting protease production are sources of carbon, nitrogen, growth factors and metal ions [1]. Besides, physical factors such as temperature, pH, aeration/agitation and incubation time also significantly affect the protease production [3]. Therefore, optimization of nutritional and physical parameters for maximum enzyme production is of utmost importance for making the industrial process cost-effective and economically viable.

In conventional 'one-variable-at-a-time' approach, the nutritional/cultural factors are optimized by changing one factor at a time, and keeping other variables constant. This approach is simplest to implement, and primarily helps in selection of significant parameters affecting the enzyme yield. However, this method is not only time restrictive, but also ignores the combined interaction(s) among various physical and nutritional parameters [4]. Conversely, the statistical response surface methodology (RSM) is a useful model for simultaneously studying the effect of several factors influencing the process of enzyme production. This also reduces the number of experiments required in growth medium optimization. Use of factorial designs and regression analyses for generating empirical models makes RSM a good statistical tool [5]. To analyze the effect of various factors in better way, a number of statistical approaches with response surface methodology are attempted for the optimization of enzyme production.

Scaling-up of protease production is also governed by aeration, agitation and dissolved oxygen tension (DOT) of fermenting medium. Agitation and aeration processes are important variables in aerobic fermentation, as oxygen availability and its proper distribution in the fermenting medium is regulatory for protease production. At flask level fermentation process, only agitation can be regulated; however, it results in limited aeration, and hence reduced protease yield. This could be due to less availability of dissolved oxygen (DO), which adversely affects the cell growth and enzyme yield [6]. Hence, bioreactors are preferred over flask level microbial fermentation for optimization of aeration, agitation and dissolved oxygen tension. Although, bioreactors provide efficient oxygen distribution in the fermenting broth, sometimes increased shear can reduce the protease yield. So, a proper ratio of agitation and aeration is required for maximization of protease production [7]. Therefore, optimal conditions for expression of high activity must be first determined in laboratory-scale followed by pilot- and industrial-level fermentors [8].

Keeping the above in view, the present study is an attempt to enhance protease production through optimization of nutritional/physical parameters by conventional (one-variable-at-a-time) and statistical approach (RSM) at shake flask level. Further, the effect of agitation, aeration and dissolved oxygen tension (DOT) on production of a psychro-thermo-alkalistable protease from *Pseudomonas putida *SKG-1 in bench-scale fermentor (under RSM optimized nutritional and cultural parameters) was also envisaged.

## Materials and Methods

### Microorganism and protease production

*Pseudomonas putida *SKG-1 (MTCC 10510) was isolated in our laboratory. This solvent and heavy metal tolerant strain is capable of producing solvent-, psychro-, thermo-, alkali-stable protease [9]. The pure culture was maintained over nutrient agar slants (pH 7.0) at 4°C.

For protease production, 250 ml sterile modified GYE (MGYE) broth [10] of pH 9.0 was taken in 500 ml Erlenmeyer flask, inoculated with 2% (v/v) mother culture of 0.8 OD (A_660_; 1 cm cuvette) containing 2.8 × 10^8 ^colony forming units (cfu) ml^-1 ^and incubated at 25°C in shaker incubator (150 rpm). The fermenting broth (5.0 ml) was periodically drawn aseptically, and growth was assessed by turbidity measurement at 660 nm. Each sample was then centrifuged at 12,000 rpm (4°C) for 10 min, and cell-free supernatant was used for protease assay.

### Enzyme assay

The protease activity was assayed by casein digestion method of Shimogaki et al. [11] at 40°C and pH 9.5 (sodium carbonate-bicarbonate buffer). One unit of protease activity is defined as the amount of enzyme required to liberate 1 μg of tyrosine min^-1^.

### Optimization of nutritional and physical parameters

In the conventional scaling-up approach, various nutritional and physical parameters were optimized by maintaining all factors at a constant level in the basal medium, except the one under study. Each subsequent factor was examined after taking into account the previously optimized factor(s). Among carbon sources fructose, xylose, lactose, sucrose, soluble starch, maltose, glycerol and carboxymethyl cellulose (CMC) were supplemented individually by replacing glucose in the basal medium. All sugars were autoclaved separately at 10 psi for 20 min, and added at 1.0% (w/v) level. For the effect of different nitrogen sources, peptone plus yeast extract in MGYE broth were replaced individually by gelatin, urea, casein, casamino acid, beef extract, yeast extract, tryptone, sodium nitrate, ammonium nitrate and ammonium sulfate at 1.0% (w/v) level. Combination of each nitrogen source with 0.5% (w/v) yeast extract (YE) was also attempted. After optimization of carbon and nitrogen sources along with their concentrations, varied levels of yeast extract (0.1-0.7% w/v) were also studied to optimize its dose. Metal cations (0.01% w/v) studied to enhance protease yield were: Ca^2+ ^(CaCl_2_), Cu^2+ ^(CuSO_4_), Mg^2+ ^(MgSO_4._), Mn^2+ ^(MnSO_4_), Fe^2+ ^(FeCl_2_), Zn^2+ ^(ZnSO_4_) and combination of Ca^2+ ^+Mg^2+ ^ions. The dose of best metal ion was optimized by its supplementation at 0.005-0.05% (w/v) concentrations. Initial pH of the medium (7.0-10.0), shaking speed (0-250 rpm), temperature (10-40°C) and time of incubation (0-72 h) were the physical parameters studied for their effect on bacterial growth and protease production.

### Statistical optimization of factors affecting protease production by RSM

Box-Behnken design (Design Expert 8.0.5) was adapted to define the nature of response surface in the experimental region, and to identify the optimal level of four most significant conventionally optimized variables, *viz*., glucose (A), yeast extract (B), MgSO_4 _(C) and pH (D). The experimental design was generated and analyzed by using statistical software Design Expert-8.0.5. The effect of each variable on enzyme production was studied at three different levels (-1, 0 and +1) with minimum, central and maximum value (Table 1), and thirty (30) experimental setups were obtained (Table 2).

**Table 1:** Experimental range and the levels of four independent variables employed in RSM in terms of actual and coded factors


**Variables**	**Levels**		
	
	**-1**	**0**	**+1**

Glucose (%, w/v)	0.75	1.00	1.25
Yeast extract (%, w/v)	0.30	0.50	0.70
MgSO_4 _(%, w/v)	0.0075	0.0100	0.0125
pH	8.80	9.00	9.20

**Table 2:** Experimental designs used in RSM studies by using four independent variables with six centre points showing observed and predicted values of protease production


**Standard Order**	**Factor A** **(Glucose %, w/v)**	**Factor B** **(Yeast extract %, w/v)**	**Factor C (MgSO_4 _%, w/v )**	**Factor D (pH)**	**Observed response (Uml^-1^)**	**Predicted response (Uml^-1^)**

**1**	0.75	0.30	0.0100	9.00	419	418.13
**2**	1.25	0.30	0.0100	9.00	457	455.63
**3**	0.75	0.70	0.0100	9.00	449	451.13
**4**	1.25	0.70	0.0100	9.00	482	483.63
**5**	1.00	0.50	0.0075	8.80	524	562.46
**6**	1.00	0.50	0.0125	8.80	540	541.96
**7**	1.00	0.50	0.0075	9.20	372	370.79
**8**	1.00	0.50	0.0125	9.20	396	394.29
**9**	0.75	0.50	0.0100	8.80	561	561.58
**10**	1.25	0.50	0.0100	8.80	617	612.58
**11**	0.75	0.50	0.0100	9.20	426	425.92
**12**	1.25	0.50	0.0100	9.20	447	444.92
**13**	1.00	0.30	0.0075	9.20	369	365.25
**14**	1.00	0.70	0.0075	9.00	415	413.75
**15**	1.00	0.30	0.0125	9.00	406	402.75
**16**	1.00	0.70	0.0125	9.00	416	415.25
**17**	0.75	0.50	0.0075	9.00	446	446.63
**18**	1.25	0.50	0.0075	9.00	479	482.13
**19**	0.75	0.50	0.0125	9.00	466	466.63
**20**	1.00	0.50	0.0125	9.00	498	501.13
**21**	1.00	0.30	0.0125	8.80	509	512.96
**22**	1.00	0.70	0.0100	8.80	513	511.46
**23**	1.00	0.30	0.0100	9.20	324	329.29
**24**	1.00	0.70	0.0100	9.20	392	391.79
**25**	1.00	0.50	0.0100	9.00	514	514
**26**	1.00	0.50	0.0100	9.00	514	514
**27**	1.00	0.50	0.0100	9.00	514	514
**28**	1.00	0.50	0.0100	9.00	514	514
**29**	1.00	0.50	0.0100	9.00	514	514
**30**	1.00	0.50	0.0100	9.00	514	514

A second order polynomial equation was used for the analysis of protease production, and the data were fitted in the equation by multiple regression procedure. This resulted in an empirical model. The model equation for analysis is as under:

(1) $$\text{Y}=\beta_{0}+\sum \beta_{\text{n}}{\text{X}}_{\text{n}}+\sum \beta_{\text{nn}}{\text{X}}_{\text{n}}^{2}+\sum \beta_{\text{nm}}{\text{X}}_{\text{n}}{\text{X}}_{\text{m}}$$

Where, Y is the predicted response, β_o _offset term, β_n _liner coefficient, β_nn _squared coefficient, β_nm _interaction coefficient, X_n _n^th ^independent variable, X_n_^2 ^squared effect and X_n_X_m _interaction effects.

For four variable systems, the model equation is as follows:

(2) $$\begin{array}{l} \text{Y}=\beta_{0}+\beta_{1}\text{A + }\beta_{2}\text{B + }\beta_{3}\text{C + }\beta_{4}\text{D + }\beta_{11}{\text{A}}^{2}+\beta_{22}{\text{B}}^{2}+\beta_{33}{\text{C}}^{2}+\beta_{44}{\text{D}}^{2}\\ \;+\beta_{12}\text{AB + }\beta_{13}\text{AC + }\beta_{14}\text{AD + }\beta_{23}\text{BC + }\beta_{24}\text{BD + }\beta_{34}\text{CD} \end{array}$$

Design-Expert software was used to obtain the coefficient of equation (2) based on data provided in Table 2. Analysis of variance (ANOVA) was used to analyze the responses under different combinations as defined by the design (Table 3).

**Table 3:** ANOVA for Response Surface Quadratic Model


**Source**	**Sum of squares**	**df**	**Mean square**	***F*-value**	**p-value**

Model	1.205E+005	14	8609.99	820.46	< 0.0001
A-Glucose	3675.00	1	3675.00	350.20	< 0.0001
B-Yeast extract	2790.75	1	2790.75	265.94	< 0.0001
C-MgSO_4_	1140.75	1	1140.75	108.70	< 0.0001
D-pH	69008.33	1	69008.33	6575.95	< 0.0001
AB	6.25	1	6.25	0.60	0.4531
AC	0.25	1	0.25	0.024	0.8795
AD	256.00	1	256.00	24.39	0.0002
BC	324.00	1	324.00	30.87	< 0.0001
BD	1024.00	1	1024.00	97.58	< 0.0001
CD	16.00	1	16.00	1.52	0.2372
A^2^	274.05	1	274.05	26.12	0.0002
B^2^	30325.24	1	30325.24	2889.76	< 0.0001
C^2^	13950.10	1	13950.10	1329.33	< 0.0001
D^2^	555.00	1	555.00	52.89	< 0.0001
Residual	146.92	14	10.49		
Lack of fit	146.92	10	14.69		
Pure error	0.000	4	0.000		
Cor total	1.205E+005	28			
**Standard deviation**	**3.24**		**R-squared**		**0.9988**
**Mean**	**465.38**		**Adjusted R-squared**		**0.9976**
**Coefficient of variation (C.V.%)**	**0.70**		**Predicted R-squared**		**0.9930**
**PRESS**	**846.24**		**Adequate precision**		**121.595**

### Bench-scale bioreactor level optimization

#### Effect of aeration

Fermentation was performed in a stirred tank bioreactor (Bioflo 110, New Brunswick Scientific Co. Inc. Edison, NJ, USA) of 3 liter capacity. The fermentor was equipped with direct drive dual Rushton style impeller, PID temperature and agitation control, probes and controller for pH and DO. For protease production, fermentation was carried out in one liter GGY broth under conventional and RSM optimized nutritional (glucose 1.25%, gelatin 2%, yeast extract 0.5%, Mg^2+ ^0.01%) and cultural (pH 8.8, 25°C, 200 rpm) conditions. The medium was inoculated (2%, v/v) with the mother culture of strain SKG-1 (0.8 OD, A_660_; 1 cm cuvette) containing 2.8 × 10^8 ^cfu ml^-1^. The aeration of culture broth was effected at different rates (0-1.5 vvm), and samples (5.0 ml) were drawn periodically at 12 h intervals. The bacterial growth was assessed by turbidity measurement at 660 nm. The sample broth was then centrifuged at 12,000 rpm (4°C) for 10 min, and cell-free supernatant was used to assay the protease activity. Change in DOT during the course of fermentation was also recorded throughout the incubation period.

#### Effect of agitation at optimized aeration

The growth and protease production was further studied by varying the agitation speed from 100 to 250 rpm at optimized aeration rate. Other experimental conditions remained the same.

### Statistical analysis

Each set of experiment was performed thrice, and all values presented here are average of three independent experiments. The standard deviation for each value is ≤ 5%.

## Results and Discussion

### Protease production

**Figure 1:**
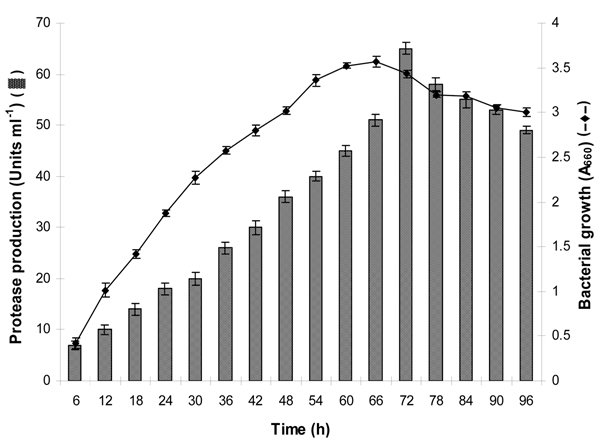
**Bacterial growth and extracellular protease production in modified GYE broth (pH 9.0) at 25°C and 150 rpm during 96 h incubation**.

The strain SKG-1 exhibited typical sigmoidal growth curve in modified GYE broth (Figure 1). After a steep exponential growth, the onset of stationary phase was at 60^th ^h onwards, and attained maximum growth and protease production at 66 and 72 h, respectively. The enzyme production initiated at 6^th ^h of bacterial growth with maximum 65 Uml^-1 ^during stationary phase at 72 h incubation, which thereafter decreased with time (Figure 1). Maximum enzyme production during stationary growth phase is in accordance with the findings of other researchers [2,10]. *Pseudomonas aeruginosa *PseA strain exhibited slow growth up to 12 h, exponential growth up to 48 h, followed by a stationary phase. The protease secretion corresponded with the growth response, and reached maximum during the late exponential/early stationary phase [3].

### Optimization of nutritional and physical parameters

#### Carbon source

Maximum protease was produced with soluble starch (71 Uml^-1^) followed by glucose (65 Uml^-1^) at 72 h incubation. Other carbon sources produced less enzyme in the following order (Uml^-1^): fructose (54) > maltose (49) > sucrose (43) > glycerol (40) > xylose (36) > lactose (33) > carboxymethyl cellulose (27) (Table 4). Although maximum enzyme was produced by soluble starch, glucose (second best) was selected for further studies due to cost consideration. Further, maximum protease (67 Uml^-1^) was produced at 1.5% glucose level though, it was merely 3% more (65 Uml^-1^) than that produced at 1.0% (w/v) glucose concentration. Therefore, 1.0% (w/v) glucose was selected as the suitable concentration for further optimization of protease production (Table 4). An inducible effect of glucose on protease production was reported by other researchers also [1,12]. Gupta and Khare [3] reported that *Pseudomonas aeruginosa *PseA produced maximum protease by CM-cellulose as the best carbon source followed by glycerol, sucrose, maltose and fructose. However, CM-cellulose was not selected for further studies rather glycerol, the second best carbon source was preferred due to economic reasons.

**Table 4:** Effect of different carbon and nitrogen sources on protease (Uml^-1^) production (A) and growth (B) of *Pseudomonas putida *SKG-1 at pH 9.0, 25°C and 150 rpm during 96 h incubation


**Nutritional** **Parameters (% w/v)**		**Incubation Time (h)**															

**Carbon sources**		**12**		**24**		**36**		**48**		**60**		**72**		**84**		**96**	
	
		**A**	**B**	**A**	**B**	**A**	**B**	**A**	**B**	**A**	**B**	**A**	**B**	**A**	**B**	**A**	**B**

Glucose	(2.5)	2	1.62	5	3.10	13	3.81	24	4.01	33	4.71	41	4.58	39	4.49	36	4.35
Glucose	(2)	4	1.21	7	2.41	15	3.42	28	4.13	42	4.51	58	4.32	56	4.18	53	4.12
Glucose	(1.5)	6	1.15	14	2.20	20	3.13	34	3.82	48	4.35	**67**	4.17	65	3.9	62	3.70
Glucose	(1)	10	1.01	18	1.87	26	2.57	37	3.02	46	3.53	**65**	3.43	63	3.28	59	3.15
Glucose	(0.5)	8	1.01	15	1.65	19	2.29	28	2.78	35	3.05	44	2.91	42	2.80	40	2.69
Glucose	(0.0)	6	0.91	13	1.65	21	2.10	27	1.75	26	1.57	23	1.41	21	1.32	19	1.18
Fructose	(1)	6	0.98	11	1.61	18	2.18	26	2.87	38	3.37	54	3.16	52	3.05	50	2.73
Lactose	(1)	00	0.46	4	0.78	10	1.14	16	1.65	21	1.83	33	1.74	31	1.64	29	1.58
Sucrose	(1)	4	0.62	9	1.05	17	2.21	28	2.96	32	3.41	43	3.29	41	3.10	38	2.84
Maltose	(1)	5	0.78	8	1.18	15	2.29	26	2.85	38	3.48	49	3.32	48	3.22	45	3.07
Soluble starch	(1)	7	0.52	19	1.12	28	1.89	42	2.91	54	3.12	**71**	2.94	69	2.93	66	2.88
CMC	(1)	00	0.42	5	0.61	9	1.11	14	1.84	20	2.59	27	2.41	27	2.38	25	2.26
Glycerol	(1)	8	0.83	14	1.03	21	1.85	30	2.57	34	2.91	40	2.78	39	2.66	34	2.40
Xylose	(1)	3	0.67	8	1.05	14	1.85	19	2.44	23	3.06	36	2.83	344	2.71	31	2.62

**Nitrogen sources**																	

Cas amino acid	(1)	3	0.75	5	1.16	7	1.98	13	2.68	17	2.96	21	2.78	20	2.71	19	2.64
Ammonium sulfate	(1)	00	0.12	00	0.31	3	0.67	5	0.88	8	0.76	8	0.67	5	0.60	4	0.48
Ammonium nitrate	(1)	00	0.11	00	0.41	00	0.61	2	0.57	4	0.40	4	0.38	2	0.26	00	0.21
Sodium nitrate	(1)	00	0.16	00	0.46	00	0.92	4	1.34	7	1.28	7	1.06	6	0.96	4	0.73
Urea	(1)	00	0.12	00	0.36	00	0.71	3	1.32	6	1.17	6	0.95	4	0.73	1	0.41
Yeast extract	(1)	3	0.94	6	1.62	8	2.90	11	3.28	13	3.51	15	3.30	14	3.18	12	3.09
Beef extract	(1)	2	0.71	5	1.05	8	1.87	9	2.24	10	2.56	12	2.43	12	2.28	10	2.21
Tryptone	(1)	4	0.79	6	1.24	11	2.10	16	2.81	19	3.15	23	2.94	22	2.80	21	2.71
Casein	(1)	1	0.72	5	1.18	9	1.92	11	2.43	14	2.78	18	2.60	17	2.56	15	2.40
Gelatin	(1)	3	0.81	8	2.05	14	2.78	22	3.27	38	3.40	57	3.28	55	3.21	51	3.18
Peptone	(1)	4	0.86	7	1.47	13	2.32	21	2.97	24	3.28	28	3.15	27	3.06	25	2.84

**Nitrogen sources with yeast extract (0.5)**																	

Casamino acid	(1)	5	1.07	11	2.13	24	2.88	31	3.07	40	3.12	51	2.86	49	2.69	47	2.51
Ammonium sulfate	(1)	1	0.26	3	0.51	4	0.96	7	1.43	9	2.14	14	1.85	14	1.79	12	1.52
Ammonium nitrate	(1)	00	0.14	1	0.25	2	0.63	6	0.94	4	1.33	6	1.07	5	0.96	5	0.81
Sodium nitrate	(1)	00	0.17	1	0.31	3	0.76	4	1.13	6	1.50	9	1.36	8	1.20	7	1.06
Urea	(1)	00	0.12	2	0.27	2	0.60	5	0.99	7	1.26	8	1.14	8	1.07	6	0.91
Peptone	(1)	10	1.01	18	1.87	26	2.57	37	3.02	46	3.53	65	3.43	63	3.28	59	3.15
Beef extract	(1)	6	1.03	13	1.84	19	2.35	24	2.61	30	2.81	34	2.65	32	2.53	29	2.48
Tryptone	(1)	5	1.11	15	1.73	26	2.42	32	2.95	39	3.20	53	3.16	51	2.98	50	2.79
Casein	(1)	4	0.92	7	1.46	12	1.99	19	2.71	24	3.06	30	2.81	28	2.65	26	2.50
Gelatin	(0.5)	7	0.63	18	1.30	31	2.43	52	3.05	88	3.26	106	3.19	103	3.10	95	3.09
Gelatin	(1)	11	1.11	27	2.05	49	2.95	81	3.45	139	3.68	171	3.58	168	3.44	164	3.26
Gelatin	(1.5)	18	1.14	45	2.12	78	3.33	121	4.41	183	4.56	210	4.49	203	4.38	191	4.24
Gelatin	(2)	29	1.23	84	2.62	115	3.84	182	4.71	239	4.96	**278**	4.87	269	4.80	251	4.71
Gelatin	(2.5)	27	1.20	76	2.64	112	3.99	171	4.92	230	5.20	264	5.14	256	5.00	239	4.82
Gelatin	(3)	9	0.97	19	1.86	42	2.53	74	3.54	128	3.96	154	3.75	149	3.60	144	3.48

#### Nitrogen source

Each organic and inorganic nitrogen source employed supported bacterial growth and protease production. However, maximum enzyme was produced with gelatin plus yeast extract (Table 4). Other nitrogen sources either alone or in combination with yeast extract produced lesser enzyme. In general, protease production was more with organic as compared to inorganic nitrogen sources (Table 4). Although, yeast extract in combination with inorganic nitrogen sources marginally increased enzyme production, it was meagre compared to complex nitrogen sources. The reduced protease production in the presence of inorganic nitrogen sources is in agreement with the findings of other researchers [3,13,14]. Complex nitrogen sources are generally required for protease production; however, the requirement of specific nitrogen source varies from organism to organism [15]. Several researchers have reported maximum protease production in the presence of complex nitrogen sources [3,16]. In the present study, gelatin (best nitrogen source) at 2% (w/v) plus yeast extract (0.5%, w/v) were most suitable for maximum (278 Uml^-1^) protease production (Tables 4 and 5). Gupta and Khare [3] found 0.6% (w/v) yeast extract most suitable for maximum protease production by *P. aeruginosa *PseA. Yeast extract not only serves as a nitrogen source, but also provides vitamins for promoting bacterial growth and enzyme production [17].

**Table 5:** Effect of yeast extract, metal ions and physical factors on protease (Uml^-1^) production (A) and bacterial growth (B) at pH 9.0, 25°C and 150 rpm during 96 h incubation


**Yeast extract and metal ions** **(%, w/v)**		**Incubation Time (h)**															

**Yeast extract**		**12**		**24**		**36**		**48**		**60**		**72**		**84**		**96**	
	
		**A**	**B**	**A**	**B**	**A**	**B**	**A**	**B**	**A**	**B**	**A**	**B**	**A**	**B**	**A**	**B**

Yeast extract	(0.0)	9	0.96	17	2.41	28	3.21	41	3.52	66	3.74	89	3.66	84	3.60	81	3.51
Yeast extract	(0.1)	14	1.08	31	2.40	52	3.40	76	3.75	93	3.84	110	3.72	108	3.65	105	3.61
Yeast extract	(0.2)	17	1.16	54	2.47	67	3.46	103	3.82	154	3.95	178	3.81	170	3.74	159	3.69
Yeast extract	(0.3)	20	1.18	65	2.49	92	3.67	152	4.21	203	4.38	219	4.20	213	4.14	208	4.07
Yeast extract	(0.4)	28	1.21	79	2.53	109	3.79	167	4.63	227	4.77	255	4.64	246	4.58	239	4.50
Yeast extract	(0.5)	29	1.23	84	2.62	115	3.84	182	4.71	239	4.96	**278**	4.87	269	4.80	251	4.71
Yeast extract	(0.6)	26	1.30	67	2.97	105	4.11	178	4.91	231	5.17	269	5.04	257	4.97	248	4.91
Yeast extract	(0.7)	21	1.39	48	3.16	92	4.50	163	5.20	227	5.35	246	5.26	241	5.18	237	5.03

**Metal ions**																	

No metal		29	1.23	84	2.62	115	3.84	182	4.71	239	4.96	278	4.87	269	4.80	251	4.71
ZnSO_4_	(0.01)	6	1.34	17	3.62	36	4.93	67	5.82	92	6.05	103	5.87	100	5.54	94	5.17
CuSO_4_	(0.01)	5	0.51	12	1.32	41	2.15	98	2.62	159	2.94	186	2.77	182	2.61	174	2.54
FeCl_2_	(0.01)	8	0.62	31	1.40	74	1.95	154	2.17	186	2.31	210	2.27	207	2.08	201	1.83
MnSO_4_	(0.01)	4	0.82	15	1.43	34	3.05	78	3.67	133	4.15	147	3.94	145	3.80	141	3.64
CaCl_2_	(0.01)	33	1.16	86	3.08	143	4.60	196	5.23	289	5.82	342	5.53	336	5.38	327	4.94
MgSO_4_ + CaCl_2_	(0.005 + 0.005)	36	1.19	92	2.97	158	4.82	209	5.34	318	5.95	377	5.76	372	5.60	364	5.23
MgSO_4_	(0.005)	36	1.23	77	2.86	159	3.90	216	5.69	297	5.91	354	5.57	349	5.21	342	5.19
MgSO_4_	(0.01)	41	1.20	106	3.10	197	4.73	304	6.07	386	6.18	**431**	6.10	428	5.83	417	5.26
MgSO_4_	(0.02)	38	1.80	81	4.13	175	5.20	286	6.22	364	6.38	378	6.15	376	6.03	371	5.86
MgSO_4_	(0.03)	35	1.62	62	3.57	139	5.14	246	6.11	327	6.15	356	5.96	352	5.81	346	5.66
MgSO_4_	(0.04)	34	1.45	66	3.40	122	4.93	212	5.88	305	5.07	321	5.82	314	5.73	306	5.43
MgSO_4_	(0.05)	27	1.40	59	2.89	108	4.76	176	5.32	264	5.73	309	5.24	301	5.10	296	5.08

**Effect of physical parameters on bacterial growth and protease production in optimized medium containing** **(gl^-1 ^distilled water): glucose, 10.0; gelatin, 20.0; yeast extract, 5.0 and MgSO_4_.7H_2_O, 0.1**																	

**Incubation Time (h)**																	

**pH**		**12**		**24**		**36**		**48**		**60**		**72**		**84**		**96**	
	
		**A**	**B**	**A**	**B**	**A**	**B**	**A**	**B**	**A**	**B**	**A**	**B**	**A**	**B**	**A**	**B**

7.0		00	0.63	00	1.73	00	2.95	00	4.38	00	4.92	00	4.78	00	4.64	00	4.51
7.5		00	0.69	00	1.83	7	3.06	19	4.57	32	5.10	38	4.85	35	4.69	31	4.32
8.0		12	0.93	21	2.40	34	3.32	48	4.87	64	5.30	87	5.14	81	5.02	78	4.85
8.5		30	1.18	76	3.04	165	4.52	213	5.77	294	6.08	311	5.84	308	5.61	304	5.22
9.0		41	1.20	106	3.10	197	4.73	304	6.07	386	6.18	**431**	6.10	428	5.83	417	5.26
9.5		5	1.08	17	3.06	48	4.26	92	5.17	135	5.50	146	5.34	143	5.21	138	5.02
10.0		00	0.25	00	0.34	00	0.21	00	0.19	00	0.16	00	0.13	00	0.12	00	0.09

**Agitation speed (rpm)**																	

50		14	0.62	26	1.44	41	2.58	58	3.06	81	3.30	97	3.16	95	3.08	93	3.01
100		27	0.93	93	2.17	139	3.86	187	4.23	249	4.95	283	4.70	279	4.40	273	4.19
150		41	1.20	106	3.10	197	4.73	304	6.07	386	6.18	431	6.10	428	5.83	417	5.26
200		53	1.46	127	3.81	261	5.48	375	6.32	**514**	6.21	509	6.03	501	5.64	492	5.17
250		46	1.30	113	4.05	254	5.72	368	6.29	491	6.14	483	5.80	476	5.64	468	5.37

**Temperature (°C)**																	

10		4	0.42	10	0.79	18	1.13	27	1.84	32	2.26	46	2.81	53	2.51	51	2.42
15		13	0.61	32	1.06	59	1.92	104	2.68	167	3.96	219	3.74	217	3.58	216	3.42
20		24	0.92	45	1.75	112	3.87	287	5.11	423	4.83	421	4.70	418	4.47	413	4.26
25		53	1.46	127	3.81	261	5.48	375	6.32	**514**	6.21	509	6.03	501	5.64	492	5.17
30		31	1.14	84	2.59	196	4.05	308	5.40	435	5.27	431	5.13	427	4.86	421	4.33
35		18	0.85	31	1.92	58	2.73	87	3.42	132	3.25	132	3.14	129	3.01	126	2.90
40		2	0.80	3	1.43	7	1.84	10	2.17	12	1.94	11	1.72	8	1.54	6	1.24

#### Metal ions

Table 5 reveals that Mg^2+ ^and Ca^2+ ^ions individually and in combination enhanced the protease production. However, maximum enzyme (431 Uml^-1^) was produced with Mg^2+ ^ions alone, and was therefore, selected for further studies. Other metal ions (Cu^2+^, Fe^2+^, Zn^2+^, Mn^2+^) reduced protease production (Table 5). Among different levels of Mg^2+ ^(0.005-0.05% w/v) employed, 0.01% (w/v) was most effective. Any deviation in Mg^2+ ^concentration from optimum 0.01% (w/v) adversely affected protease yield (Table 5). Our results are in accordance with the findings of other researchers, who also reported Mg^2+ ^as the best metal ion supplement for protease production and bacterial growth [18]. Rahman et al. [17] also reported enhanced protease production in the presence of Mg^2+ ^by *P. aeruginosa *strain K. The stimulating effect of CaCl_2 _was reported by Mabrouk et al. [19]. They attributed this effect to the stabilizing nature of CaCl_2 _on alkaline protease.

#### Initial pH

The organism was able to grow in the selected pH range (7.0-10.0), but protease production was restricted to pH range 7.5-9.5 only. At pH 9.0, maximum protease (431 Uml^-1^) was produced under optimized nutritional conditions. Any deviation in pH from optimum 9.0 adversely affected the bacterial growth and enzyme production. At pH 7.0 and 10.0, protease production was not detected (Table 5). The alkaline pH optimum reveals alkaliphilic nature of strain SKG-1. Joshi et al. [20] also reported maximum protease yield at pH 9.0 by *B. cereus *isolate. *Bacillus *sp. strain APP1 was able to grow well in pH range 5.0-12.0, but produced protease maximally at pH 9.0 [21]. Other researchers have reported maximum alkaline protease production at pH 7.0-7.5 also [3,13,22]

#### Agitation

Shaking of cultures significantly affected protease production, which was maximum (514 Uml^-1^) at 200 rpm. Any change in agitation speed decreased enzyme production, and produced 97, 283, 431 and 49U protease ml^-1 ^at 50, 100, 150 and 250 rpm, respectively (Table 5). Presumably at 200 rpm agitation, the aeration of culture broth increased optimally, which enhanced the supply of dissolved oxygen and uptake of nutrients to the bacterial cells. The decreased enzyme production at > 200 rpm was perhaps due to denaturation of proteases caused by the mechanical damage.

Shaking of aerobic bacterial culture is one of the most decisive factors for growth and protease production, as agitation maintains proper oxygen supply and mixing of growing cells. Oxygen transfer into bacterial cells in aerobic fermentation process strongly affects growth and enzyme production by affecting the metabolic pathways and fluxes [23]. Several other researchers also reported maximum protease production at an agitation speed of 200 rpm [22]. Gupta and Khare [3] reported maximum protease production by *P. aeruginosa *PseA at 250 rpm.

#### Temperature

In any bioprocess, specific temperature requirement and its regulation is one of the most critical parameters. Strain SKG-1 was able to grow and produce protease in complete temperature range (10°- 40°C) of study with maximum production at optimum 25°C. Temperature higher or lower than optimum, reduced the bacterial growth, thereby steady decrease in enzyme production (Table 5). The order of enzyme production at other temperatures was (Uml^-1^): 10°C (53) < 15°C (219) < 20°C (423) < 25°C (514) > 30°C (435) > 35°C (132) > 40°C (12).

The ability of strain SKG-1 to grow at wide temperature range of 10°- 40°C with optimum at 25°C revealed its psychrotrophic nature. Only few psychrotrophic bacterial isolates have been reported for thermostable alkaline protease production. Jackman et al. [24] reported heat stable protease production at 25°C from psychrotrophic pseudomonads. A psychrotrophic *Exiguobacterium *sp. SKPB5 produced alkaline protease at 30°C with maximum protease activity at 50°C [25].

#### Incubation time

**Figure 2:**
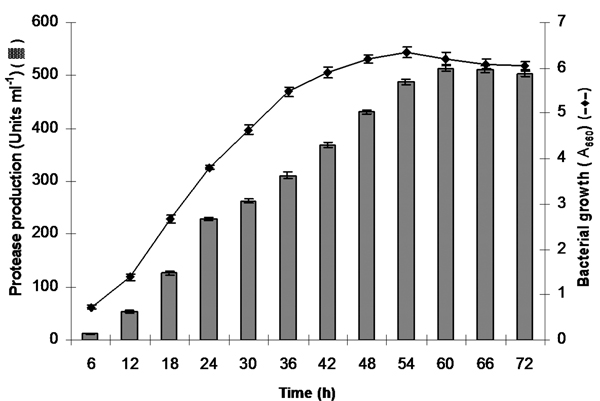
**Protease production and growth response of *P. putida *SKG-1 in finally optimized medium containing (gl^-1 ^distilled water): glucose, 10.0; gelatin, 20.0; yeast extract, 5.0 and MgSO_4_, 0.1 at initial pH 9.0, 25°C and 200 rpm during 72 h growth**.

The bacterial growth and protease production were in harmony up to 54 h under optimized nutritional and cultural conditions. Bacterial growth was in the exponential phase up to 54 h; thereafter entered the stationary phase. Whereas, enzyme production reached maximum (514 Uml^-1^) during early stationary phase at 60 h; thereafter remained nearly constant up to 72 h fermentation (Figure 2). Our findings are in accordance with the results of several other researchers [3,10]. Zeng et al. [26] reported maximum protease production of only 45 Uml^-1 ^at 10°C from *Pseudomonas *sp. strain DY-A during the late stationary phase of growth.

### Statistical optimization of factors affecting protease production by RSM

The response surface methodology (RSM) is widely applied by many researchers to optimize alkaline protease production from several bacteria and fungi. However, there is no report on statistical optimization of alkaline protease by *Pseudomonas putida*. Interactive effects of the most important conventionally optimized factors, *viz*., glucose, yeast extract, MgSO_4 _and pH were examined by RSM using Box-Behnken design. Analysis of variance (ANOVA) yielded the following regression equation in terms of the protease levels produced (Y) as a function of glucose (A), yeast extract (B), MgSO_4 _(C) and pH (D):

$$\begin{array}{l} \text{Y}=514+17.50\times \text{A}+15.25\times \text{B}+9.75\times \text{C}-75.83\times \text{C}+6.50\times \\ {\text{A}}^{2}-63.37\times {\text{B}}^{2}-46.37\times {\text{C}}^{2}-9.25\times {\text{D}}^{2}-1.25\times \text{AB}-0.25\times \\ \text{AC}-8.0\times \text{AD}-9\times \text{BC}+16\times \text{BD}+2.0\times \text{CD} \end{array}$$

Table 2 shows predicted responses of Box-Behnken design on the basis of above polynomial equation. This regression equation was assessed statistically for analysis of variance (ANOVA), and the results are predicted in Table 3. ANOVA of regression model demonstrated the determination coefficient (R^2^) 0.9988, which means 99.88% variability in the response could be explained by this model. The R^2 ^value is always between 0 and 1.0. The model is stronger and predicts better response when R^2 ^value is closer to 1.0 [5]. The value of the adjusted determination coefficient (adjusted R^2^) is 0.9976. This higher value of adjusted R^2 ^indicates greater significance of the model. A very low value of coefficient of variation (C.V., 0.70%) indicates better precision and reliability of the experiments executed. The adequate precision value of 121.595 measures signal to noise ratio, and a ratio > 4.0 is desirable. In this case, higher ratio indicates an adequate signal, and also proves that model can be used to navigate the design space.

The F- value of 820.46 in Table 3 implies that the model is significant. There is only 0.01% chance that a "model F- value" so large could occur due to noise. ANOVA analysis also indicated that the model term linear glucose (P < 0.0001), yeast extract (P < 0.0001), MgSO_4 _(P < 0.0001), pH (P < 0.0001), quadratic glucose A^2 ^(P < 0.0002), yeast extract B^2 ^(P < 0.0001), MgSO_4 _C^2 ^(P < 0.0001), pH D^2 ^(P < 0.0001) and four interaction terms were significant. Smaller the P-value, more significant is the corresponding coefficient. The P < 0.0500 indicates that model terms are significant.

**Figure 3:**
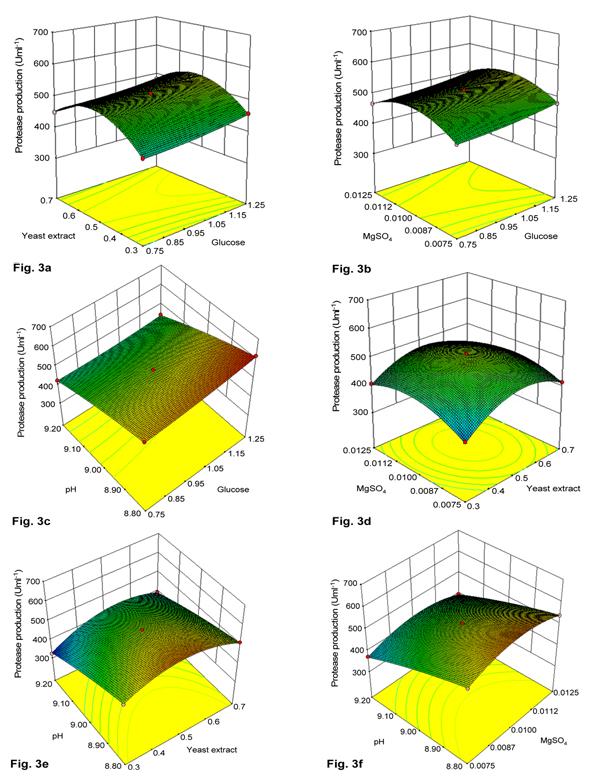
**Response-surface curve of alkaline protease production by *Pseudomonas putida *SKG-1 showing mutual interactions between (a) glucose and yeast extract, (b) glucose and MgSO_4_, (c) glucose and pH, (d) MgSO_4 _and yeast extract, (e) pH and yeast extract, (f) pH and MgSO_4_**. Other variables, except for two in each figure, were maintained at zero level in coded units.

The 3D response surface plots and two dimensional contour plots were used to understand the interaction effects of medium components and optimum concentration of each component required for maximum protease production. Response surface curves for variation in alkaline protease yield were constructed, and are depicted in Figure 3. In each set, two variables varied within their experimental range, while the other two variables remained constant at zero level.

Figure 3a depicts the production of alkaline protease with respect to glucose versus yeast extract. From the interaction response of glucose with yeast extract, protease yields increased with increasing glucose and yeast extract concentration up to 1.25% and 0.5%, respectively. The enzyme activity decreased at > 0.5% yeast extract concentration. However, the response curve did not show curvature, rather it was flattened. This suggested a demand for higher concentration of glucose. Figure 3b represents the interaction effect of glucose and MgSO_4 _on production of alkaline protease. With an increase in glucose (0.75-1.25%, w/v) and MgSO_4 _(0.0075-0.01%, w/v) concentration, the protease yield increased. Thereafter, an increase in MgSO_4 _concentration up to 0.0125% (w/v) resulted in decreased protease production. The optimal values for glucose and MgSO_4 _(w/v) were 1.25 and 0.01%, respectively.

Figure 3c reveals that maximum protease was produced at higher level of glucose (1.25%) and slightly lower alkaline pH (8.8) in the design range. This accorded a run number of 10, which is considered as the optimal condition of test variables. Table 2 shows that maximum protease of 617 Uml^-1 ^were produced at pH 8.8 (-1 in coded unit) and glucose at 1.25% (+1 in coded unit). Figure 3d depicts the interaction of two variables, *viz*., MgSO_4 _and yeast extract on protease production. The protease production increased with increasing concentration of MgSO_4 _(0.0075-0.01%, w/v) and yeast extract (0.3-0.5%, w/v). Further increase in their concentrations resulted in decreased alkaline protease production.

Figure 3e illustrates the interaction effect of pH (8.8-9.2) and yeast extract (0.3-0.7%) on alkaline protease production. Maximum enzyme units were produced with 0.5% yeast extract and pH 8.8. Further increase in pH and any deviation in yeast extract concentration from optimal, decreased the enzyme production. The effect of pH and MgSO_4 _is shown in Figure 3f. The response curve analysis indicated that protease production decreased with increase in pH from 8.8 to 9.2, and increased with enhanced concentration of MgSO_4 _up to 0.01% (w/v). Further increase in MgSO_4 _concentration above 0.01% caused decreased enzyme yield.

The above optimized results (Table 2 standard order 10) concerning four variables were finally verified by again performing the batch shake flask experiment. The maximum experimental alkaline protease production of 617 Uml^-1 ^was very close to 612 Uml^-1 ^predicted by Box-Behnken design with 1.25% glucose, 0.5% yeast extract, 0.01% MgSO_4 _and pH 8.8. Thus, under optimized conditions, the protease yield increased from 514 units in conventional optimization trial to 617 Uml^-1 ^using RSM at 60 h incubation. This proved that response surface methodology is a slightly better optimization approach as compared to conventional "one-variable-at-a-time" method in terms of improved protease yield in less time, resource and expenditure. Our findings are in agreement with the results of other researchers who have also reported RSM a better approach for enhanced protease production [4,5,16]. Reddy et al. [27] reported a 2.3 folds increase in alkaline protease production using Plackett-Burman and Response surface methodology by *Bacillus *sp. RKY3. Anbu et al. [28] obtained a good correlation coefficient of 0.9996 using Box-Behnken design and alkaline protease production of 112.90 Uml^-1 ^by *Shewanella oneidensis *MR-1 strain through response surface optimization.

### Bench-scale bioreactor optimization

#### Effect of aeration

**Figure 4:**
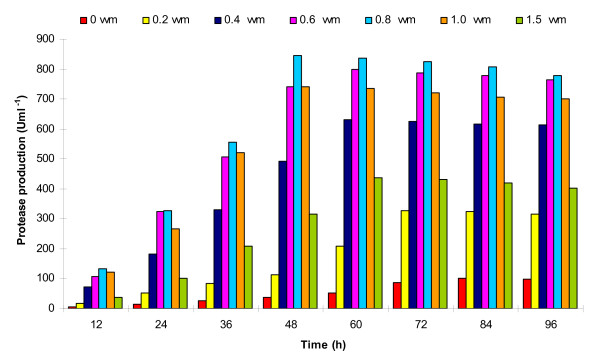
**Effect of different aeration rates (0-1.5 vvm) at constant agitation speed (200 rpm) on protease production**.

**Figure 5:**
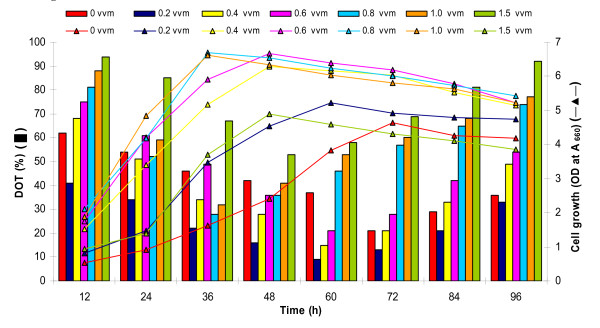
**Effect of different aeration rates (0-1.5 vvm) at constant agitation speed (200 rpm) on bacterial growth and change in DOT profile**.

In this set of experiment, the bacterial growth and protease production were studied at a fixed agitation speed of 200 rpm and variable aeration rates of 0-1.5 vvm (Figure 4). The enzyme production was drastically low at aeration rates of 0 vvm (102 Uml^-1 ^at 84 h) and 0.2 vvm (326 Uml^-1 ^at 72 h). However, at 0.4 and 0.6 vvm, the protease production was 630 U and 798 Uml^-1^, respectively at 60 h incubation. Further increase in aeration rate to 0.8 vvm produced maximum protease of 846 Uml^-1 ^at just 48 h fermentation. Still higher aeration rates of 1.0 and 1.5 vvm were detrimental for protease production, and resulted in reduced 741 Uml^-1 ^in 48 h and 436 Uml^-1 ^in 60 h, respectively (Figure 4). Bacterial growth pattern (Figure 5) was similar to protease production (Figure 4), which was very slow at lower aeration rates, and increased with increasing rate of aeration approaching maximum at 0.8 vvm. The DOT was initially 100% at 0 h, which reduced rapidly with increasing bacterial growth. The DOT was inversely related with the bacterial growth, i.e., at maximum exponential bacterial growth, the DOT was minimum and started increasing after the onset of stationary growth phase (Figure 5).

Under RSM optimized conditions, protease production was maximum at 60 h incubation, while it was highest in bioreactor trial at 48 h, thereby led to significant time saving of 12 h. Optimization of aeration rate at fixed agitation speed of 200 rpm indicated that oxygen supply to bacterial cell mass is a critical parameter for enzyme production under aerobic fermentation process. This could be achieved by optimizing the agitation speed and maintaining proper aeration; however, an appropriate balance of agitation and aeration is imperative to avoid any mechanical damage to bacterial cells which can reduce the enzyme yield. From the results of aeration optimization, it can be inferred that a direct correlation existed between bacterial growth and protease production. The reduction in DOT followed a similar pattern as experienced in previous experiment. It reduced rapidly during exponential growth phase, and rose again on commencement of stationary phase onwards (Figure 5). Rao et al. [29] reported highest protease production of 238.77 Uml^-1 ^by a *Beauveria bassiana *isolate on 6^th ^day of fermentation in a 5 litre stirred tank bioreactor at 150 rpm and 0.6 vvm aeration. Maximum protease of 340 Uml^-1 ^by *Bacillus licheniformis *NCIM-2042 was reported at aeration and agitation rates of 3 vvm and 200 rpm, respectively [7].

#### Effect of agitation at optimized aeration

**Figure 6:**
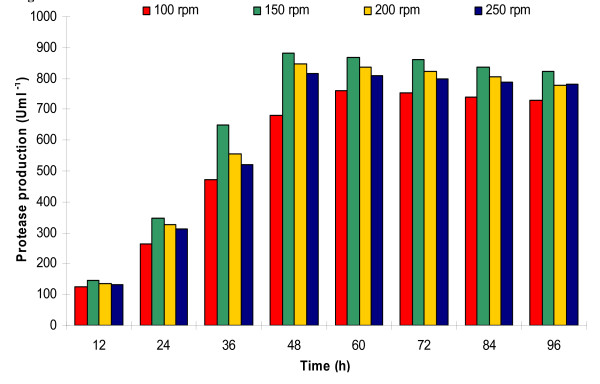
**Effect of different agitation speeds (100-250 rpm) at constant aeration rate (0.8 vvm) on protease production**.

**Figure 7:**
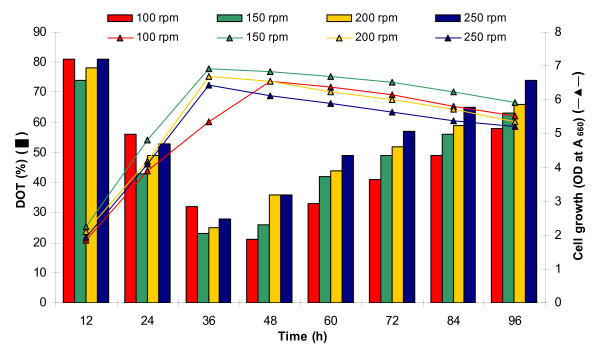
**Effect of different agitation speeds (100-250 rpm) at constant aeration rate (0.8 vvm) on bacterial growth and change in DOT profile**.

**Figure 8:**
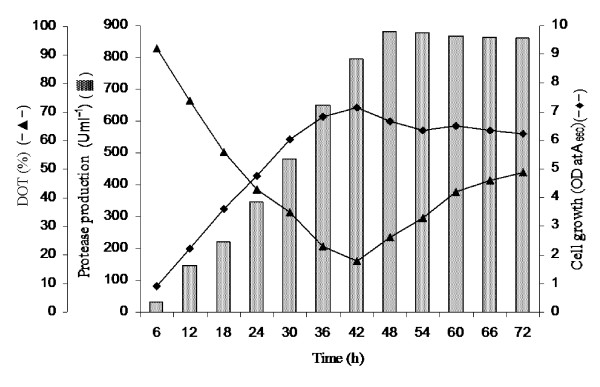
**Pattern of bacterial growth, enzyme production and DOT change in GGY broth at 150 rpm and 0.8 vvm aeration at pH 9.0 and 25°C**.

After optimization of suitable aeration rate (0.8 vvm), we attempted to optimize agitation speed (100-250 rpm) for studying its effect on protease yield and the results are depicted in Figure 6. The enzyme production enhanced with increase in agitation rate, which was maximum at 150 rpm within 48 h of incubation. The order of protease units produced at 250, 200 and 150 rpm were (Uml^-1^): 817 < 846 < 882 during 48 h fermentation. However, further decrease in agitation speed to 100 rpm reduced the enzyme yield only to 761 Uml^-1 ^at extended 60 h incubation (Figure 6). Throughout the study, the DOT started declining concomitantly with increase in bacterial growth, and reached minimum when bacterial growth was maximum. After commencement of stationary phase, the DOT again increased slowly (Figure 7). At optimized fixed aeration rate of 0.8 vvm and 150 rpm agitation, the DOT initially declined to 92% at 6 h incubation. It further declined sharply with increasing bacterial biomass, and approached minimum (18%) at 42 h, followed by slow increase to 49% at 72 h incubation (Figure 8). Thus, the protease production enhanced by ~43% (882 Uml^-1^) at bench-scale bioreactor level during 48 h incubation compared to the conventional and RSM optimization at flask level (617 Uml^-1^) during 60 h batch fermentation.

It is evident from the findings that an optimum agitation of fermenting broth is necessary for proper oxygen supply to bacterial cells. However, above the optimum speed it may damage the cells or change the cell morphology, which leads to reduced protease yield. Maximum protease production at 300 rpm has also been reported by Potumarthi et al. [7] in a stirred tank bioreactor. Any deviation in agitation speed to 200 or 400 rpm reduced the protease yield. They concluded that mixing is important for maximal protease production by optimizing both agitation and aeration for better oxygen mass transfer rate. This results in better product formation through avoiding/minimizing any mechanical damage to the bacterial cells. Many other researchers also reported the optimum agitation speed range of 150-300 rpm for protease production from different isolates [30-32]. It may, therefore, be inferred that a proper ratio of agitation and aeration is mandatory for appropriate oxygen transfer to bacterial cell mass, and also to minimize the shearing effect for maximum growth and protease production.

## Conclusions

The optimization of alkaline protease production from *Pseudomonas putida *is being reported by conventional as well as statistical response surface methodologies. A 7.9 folds (from 65 to 514 Uml^-1^) increase in protease production was evident with optimized nutritional (glucose 1%, gelatin 2%, yeast extract 0.5%, Mg^2+ ^0.01%) and cultural (pH 9.0, 25°C, 200 rpm) conditions during early stationary phase at 60 h fermentation employing conventional method of optimization. Whereas, the response surface methodology enhanced the protease production to 9.5 folds (617 Uml^-1^) by further optimizing the glucose concentration to 1.25% (w/v) and pH to 8.8. The optimization of process parameters by RSM proved it a time/resource saving and efficient method. Although, it provided better insight of interactions among the parameters that affect enzyme production, development of a better statistical tool is a constant endeavor. Further optimization of agitation (150 rpm) and aeration (0.8 vvm) rates at bench-scale bioreactor level enhanced the enzyme production by ~43% (882 Uml^-1^) at 48 h fermentation. It not only enhanced the protease yield, but also led to a significant time saving of 12 h. Furthermore, this is the first report on such a high yield of solvent and psychro-thermo-alkali-stable protease from a solvent tolerant psychrotrophic bacterial strain.

## Competing interests

The authors declare that they have no competing interests.

## Authors' contributions

1. SKS carried out the research work and drafted the manuscript.

2. SKS was involved in data processing and manuscript preparation.

3. VRT was involved in revising the manuscript critically for important intellectual contents.

4. SKK was involved in data verification and designed the optimization experiment.

5. SKG has designed the experiment(s), contributed substantially to analysis and interpretation of data and has given final approval of the version to be published.

All authors read and approved the final manuscript.
